# Developing a public-facing tool to monitor inclusion of special populations in clinical research

**DOI:** 10.1017/cts.2025.48

**Published:** 2025-03-17

**Authors:** Wendy K.K. Lam, James Goodrich, Sydney Sullivan, Keisha Bentley-Edwards, Dwight Koeberl, Devon Noonan

**Affiliations:** 1 Integrating Special Populations Core, Duke Clinical & Translational Science Institute, Durham, NC, USA; 2 Duke Office of Clinical Research, Duke University School of Medicine, Durham, NC, USA; 3 Duke School of Nursing, Durham, USA

**Keywords:** Special populations, clinical research enrollment, monitoring, health equity, inclusion in research

## Abstract

Clinical trials have provided evidence for determining treatment effectiveness. However, clinical trial participants have been underrepresented by diverse and special population groups (e.g., younger and older adults, different races/ethnicities), contributing to disparities in our understanding of diseases and treatments in all those affected. Addressing these disparities in clinical trial participation would be critical to achieving health equity in the USA and beyond. To assess enrollment inclusivity in clinical research at a large academic medical center in the southeast, we used administrative information to develop a snapshot of clinical research participation by age, sex, race, ethnicity, and rurality that was accessible to the public. We compared research enrollment statistics with relevant geographic benchmarks (county, state, and national) from the 2020 US Census. Comparisons revealed 1) over-participation by females relative to county, state, and national benchmarks; 2) under-representation of Black/African Americans relative to county, but higher relative to state and national, levels; and 3) underrepresentation of Hispanic/Latino and Asian groups. The ISP Snapshot has promoted accountability and transparency in this institution’s efforts toward health equity. The process has highlighted the need to update and standardize use of outdated categories (e.g., binary gender, rural status) that limit accurate reporting.

## Introduction

Clinical trials in the USA have historically failed to enroll participants who reflect the full diversity of the population with regard to age, race, ethnicity, sex, and lifespan transitions (e.g., pregnant and lactating people). This underrepresentation in clinical trial enrollment has led to an incomplete understanding of health conditions and of how treatments affect different groups, in turn contributing to health inequities [1–3]. Legislation enacted in 1993 and 2016 [4,5] led to policies from the NIH [6] and FDA [7,8] to promote inclusion of special populations underrepresented in clinical research, including women, racial and ethnic minorities, and all ages across the lifespan [1]. These policies have increased accountability for inclusive enrollment and analytic plans in applications for funding, and have increased reporting requirements for ongoing studies. The NIH and FDA have required researchers to report on enrollment demographics and started publishing reports of this information for different subpopulations for completed studies across their portfolios [6,9–12]. Even with these policies in place, however, clinical trials have remained underrepresented by special population groups [1,13].

Increasing diversity in clinical trials has involved a multi-stakeholder approach with buy-in and actions from not only funders and regulators but also from institutions, research sites, investigators, and the community. An institution’s diversity and research inclusion initiatives could be strengthened with a better understanding of current levels of representation and disparities at the institution. The National Institutes of Health Clinical and Translational Science Awards (CTSA) Network has required hubs to increase innovation, access, and research capacity toward integrating special populations in a measurable way. Monitoring progress toward increased diversity in research has been recommended to bring accountability at an institutional level.[14,15].

### Context and contributions

To inform our mission of increasing research capacity for integrating special populations in clinical research, the Duke CTSA Integrating Special Populations (ISP) Core sought to understand the current state of enrollment inclusivity. Feedback from stakeholders – administrative and operational leadership, researchers, and members of our community advisory board – made clear the importance of making this information available. Previous work by Langford and colleagues used institutional research data to assess inclusion of special populations in research at their institution [16]. These CTSA investigators used institutional administrative data to profile the spectrum of clinical research and basic demographics to describe inclusion of special populations. The study did not report race or ethnicity of enrollees, though acknowledged the importance of this information.

We sought to develop a snapshot of demographic characteristics of clinical research participants that would be updated regularly to monitor inclusion of diverse and special populations in research. To develop a beta version of our snapshot of inclusion, we first met with key stakeholders who managed protocol and enrollment information at Duke Health, including Directors of the Duke Health Institutional Review Board (IRB) and Duke Office of Clinical Research (DOCR) to present the request and outline limitations of the current enterprise clinical research management system (CRMS) in providing such detailed reports. Our CRMS, known as OnCore, has been set up for study teams to capture key demographic data for Duke clinical research participants, including age, sex, race, ethnicity, and geographic location, with oversight procedures for reporting compliance by study teams [17]. CRMS vendors have offered summary reports to track enrollment. However, these have not provided the information needed to adequately monitor enrollment demographics across an institution’s clinical research enterprise and are not publicly accessible.

To address these limitations, our ISP team developed a plan to retrieve administrative information and enrollment demographics, identify relevant population benchmarks, and disseminate the information via data visualizations accessible to the public. Providing information on enrollment demographics at our institution has informed our efforts to promote integration of special populations into research. Identification of variables and definitions for the ISP Snapshot has facilitated changes based on directives for reporting. Awareness of these updates highlights other potential ways to assist researchers in developing inclusive target enrollment and reporting plans.

### Study aims

The aims of this study are two-fold:

1. To develop a snapshot of demographic characteristics of participants enrolled in Duke clinical research protocols that is accessible to the Duke community and broader public; and
2. To compare the demographic characteristics of Duke clinical research participants with representation in relevant population benchmarks.

We present results of these aims while describing our process. Discussion includes a review of key lessons learned to inform other institutions. We describe current status and plans for next steps to monitor research enrollment for quality improvement in support of achieving health equity in clinical research.

## Methods

### Creating a dataset of clinical research enrollment

This study was reviewed and deemed exempt by the Duke Health IRB (Protocol no. Pro00107175) as no personal health information was analyzed. To obtain clinical research enrollment information, we worked with DOCR, which manages and oversees Duke’s CRMS, OnCore, deployed for use throughout Duke Health research. This web-based CRMS has provided a comprehensive management of the clinical trial lifecycle, including participant enrollment, institutional reports, and integration with other enterprise-wide systems such as the IRB, electronic health records, and billing systems. Duke Health transitioned to use of OnCore for prospective enrollment reporting during May 2018; historical protocol information was loaded in from as early as 1983. Currently, all human subject research studies with protocols reviewed and approved by the Duke Health IRB have been entered into OnCore. Study teams have been required to record enrollment and follow up visit information for individual participants for most IRB-approved studies. Some international studies, non-interventional studies, and survey/focus group/observational studies enter study accrual information in summary form at routine intervals (e.g., quarterly summary), with no individual participant information. Oversight is provided to ensure regular compliance with entry of study and participant encounters.

### Data

We requested a report of participant demographic information for protocols of any status (abandoned, closed to accrual, IRB study closure, open to accrual, and suspended) with individual participant encounters entered in OnCore through March 25, 2021. This inclusive approach was chosen for our preliminary snapshot to understand the information available before filters were applied. The key variables of interest included age, sex, race, ethnicity, and rural status of participants based on zip code. These variables have been described below.

### Analysis

DOCR provided an extract of requested OnCore system data on a secure server, which we imported into SAS software to review and prepare for analyses. The data snapshot included more research participations (i.e., enrollment in a study) than unique participants. Because our focus was on demographics of study enrollment (vs. participants), we used research participations as the unit of interest, meaning individuals participating in more than one study were counted for each protocol enrolled. Frequencies and percentages were calculated to summarize the proportion of participations represented by different population demographic groups.

### Variable definitions

Definitions for the variables of interest for the snapshot have been described below.

*Age*. We used age of participant at the time of enrollment and accrual into a given study protocol, a continuous integer value. Of 285,209 study participations, 35 were removed as outliers (i.e., negative values and values over 100 years of age), and 2,635 did not report age at accrual (< 1% missing). We grouped accrual age into categories: ages 65 to 100, ages 27 to 64, ages 18 to 26, ages 5 to 17, under the age of 5, and not reported.

*Sex*. Participant biological sex has been indicated in OnCore as Females, Males, or Unknown. Twelve (*n* = 12) records were missing this datum (< 1% missing).

*Race*. OnCore has allowed multiple selections of race: 1) American Indian or Alaska Native, 2) Asian, 3) Black or African American, 4) Native Hawaiian or Other Pacific Islander, 5) White, 6) 2 or more races, and 7) Unknown. The value “unknown” represented a significant fraction (6.4%, *n* = 18,197); however, no values were missing. We created a grouped value “reported two or more” to encompass “2 or more races” and any combination of any two or more of the available seven OnCore selections. Race was recorded independently of ethnicity.

*Ethnicity*. OnCore has recorded participant ethnicity independently of race, as Hispanic, non-Hispanic, or reported Unknown. Less than 1 percent of records had a missing value (*n* = 105, < 0.05%).

*Rurality of zip codes*. Participant’s zip code was recorded in OnCore to indicate residential location. Rural status was determined by the current Rural-Urban Commuting Area (RUCA) codes made available by the US Department of Agriculture [18]. RUCA codes classify US census tracts into 10 codes (1–10) by population density, daily commuting, and urbanization. Codes are updated after each US Census survey for changes in census tract reconfigurations and population movement. We used the zip code approximation of the census tract-based, primary RUCA codes to categorize zip codes as urban or rural. More specifically, a primary RUCA code of 1, 2, or 3 was considered non-rural/metro and codes 4 through 10 as rural/non-metro.

### Identifying relevant benchmarks

Benchmark standards to gauge representativeness should include population demographics and health condition prevalence. Because our snapshot aggregates protocols across therapeutic areas, we focused on population demographics. Population-based benchmarks were selected from 2020 Census data to compare enrollment inclusion rates with representation at the county [19], state [13], and national level [20,21] in which DUHS is located.

### Creating snapshot visualizations

To make enrollment demographics accessible to a wide audience, we translated the snapshot statistics into data visualizations (Tableau Desktop, Datawrapper), in consultation with Duke’s Center for Data Visualization and publically available guidance. We then presented the visualizations to Duke and community stakeholders to gather feedback on clarity and usefulness of the snapshot information to inform revisions for the final version.

## Results

### Composition of demographic enrollment across Duke clinical research

Overall, the population of Duke clinical research participants included 195,859 unique individuals, who represented 285,209 study enrollments in 5,038 protocols and 177 departments within DUHS entered into OnCore from 2000 to March 25, 2021. Of the 5,038 total protocols, 4,012 (79.6%) were closed to accrual, and 1,026 (20.4%) were actively recruiting participants at the time the preliminary dataset was generated.

### Inclusion of special populations in Duke clinical research

The preliminary snapshot showed that Duke’s overall clinical research participant population through March 2021 included 58.5% females, 30.2% minority (non-White) racial or ethnic groups; 25.4% older adults (over 65 years of age), 12.7% pediatric populations (under 18 years of age), and 17.4% rural residents.

### Benchmark comparisons

To assess the relevance of geographic location as a benchmark comparison for Duke Health clinical research participants, we used zip code to determine participants’ reported address. Based on zip codes reported in OnCore, 91%, 75%, and 24% of Duke Health clinical research participants live in the USA, the state of North Carolina, and Durham County, NC, respectively. Nearly 9% of participants had zip codes with undetermined locations.

Compared with benchmarks from the 2020 Census data, Duke’s clinical research participation indicates an overrepresentation of females at the county, state, and national levels; underrepresentation of Black/African Americans relative to county statistics, but higher representation relative to the state (NC) and the US However, research participation indicates an underrepresentation of Hispanic/Latino and Asian groups, and of children (< 18 years old). The demographic characteristics of Duke’s clinical research enrollment are summarized in Table 1.

**Table 1.** Characteristics of participants enrolled in Duke clinical research

Duke Clinical Research Enrollment Characteristics (through 3/25/21)	N	Percent (%)
Total number of unique participants	195,859	–
Participations (enrollments into a study)	285,209	**–**
Total number of protocols	5,038	**–**
**Demographics of Enrolled Participants**		
Sex		
Females	166,787	58.5
Males	117,613	41.2
Unknown*	797	0.3
Not Reported**	12	<0.005
Race		
White	189,521	66.4
Black or African American	63,998	22.4
Asian	6,228	2.2
Reported Two or More	5,372	1.9
American Indian or Alaska Native	1,607	0.6
Native Hawaiian or Other Pacific Islander	286	0.1
Unknown†	18,197	6.4
Ethnicity		
Non-Hispanic	226,078	79.3
Hispanic or Latino	12,163	4.3
Unknown^†^	46,863	16.4
Not Reported^‡^	105	<0.05
Rural Status of Zip Codes		
Non-Rural Zip Codes (codes 1-3)	210,821	73.9
Rural Zip Codes (codes 4-10)	49,673	17.4
Zip Code not reported in OnCore	24,498	8.6
Zip Code not found in the file of RUCA codes	217	<0.1
Age at Enrollment		
Ages 65 to 100	72,332	25.4
Ages 27 to 64	149,749	52.5
Ages 18 to 26	24,275	8.5
Ages 5 to 17	17,646	6.2
Under 5	18,537	6.5
Not Reported	2,635	0.9
Outliers^††^	35	<0.05

† Unknown = reported value of “unknown.”

‡ Not Reported = missing value in variable field.

†† Age outliers = negative number; age>100 years. RUCA = Rural-Urban Commuting Area.

### Designing the final ISP dashboard

The final version of the ISP Snapshot was redesigned and updated through October 2023, with quarterly updates started in 2024 for periodic dashboard monitoring. During the development of the ISP Snapshot, DOCR created a Demographics Enrollment Dashboard using Tableau, which updates daily and feeds into an administrative Scorecard for institutional leadership [17]. While the DOCR dashboard was made with expanded access beyond OnCore users, viewing the DOCR dashboard has been restricted to Duke users directly connected to the Duke network with secured identification.

**Figure 1.**
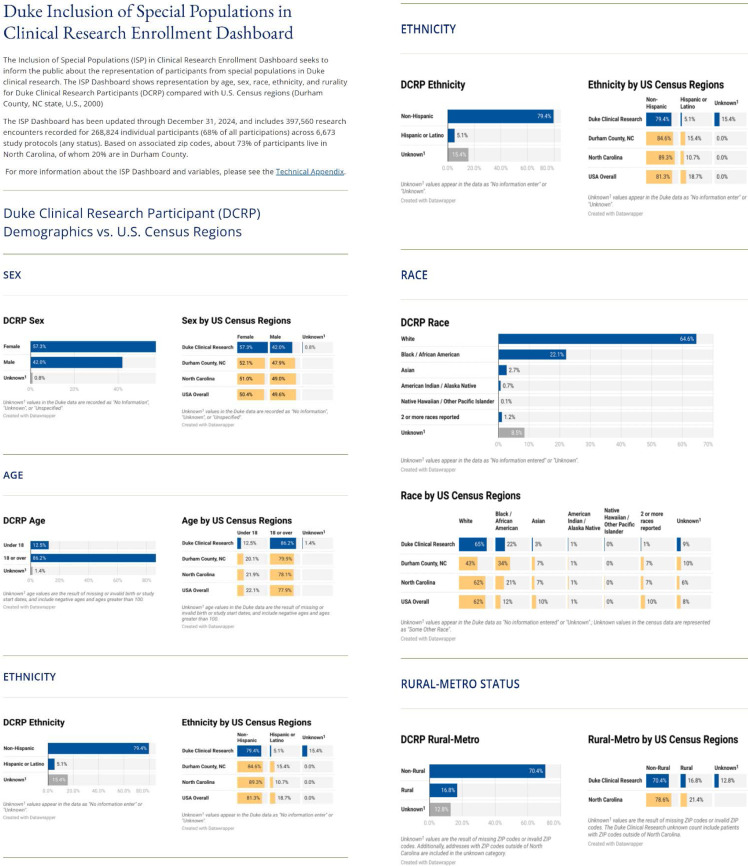
Inclusion of special populations in clinical research enrollment dashboard.

To ensure accessibility of enrollment information to the public, particularly those underrepresented in research, we prioritized accessibility, interpretability, and transparency of information for the Duke community, stakeholders, and the general public. We updated the ISP Dashboard to present only cleaned OnCore data (i.e., reviewed for outliers, invalid responses, etc.) and privacy protections. The updated ISP Dashboard has been presented in Figure 1 and has been posted on the CTSI website (https://ctsi.duke.edu/ispdashboard) [22] with the Technical Appendix describing data cleaning and variable definitions.

## Discussion

The aims of this study were to (1) develop a snapshot of enrollment demographics of special populations in Duke clinical research, accessible to the Duke Community and to the broader public, and (2) compare enrollment demographics with relevant population benchmarks. The current version of the ISP Dashboard of Inclusion in Clinical Research, with benchmarks, has been posted on the Duke CTSI website. (https://ctsi.duke.edu/ispdashboard/) [22].

Gaining a clearer picture of the current state of enrollment and inclusion of special populations in research helped focus the ISP programming activities to help address disparities in enrollment. For example, in response to the underrepresentation of Hispanic/Latinos, we gathered translation resources for Duke research teams, with approved translation and interpretation vendors and their pricing. Stakeholder feedback sessions and presentation to the CTSA Inclusion Across the Lifespan Executive Committee [23] highlighted a broader range of potential uses for the snapshot, including providing ongoing updates now available in the current ISP dashboard. CTSA hubs further expressed interest in creating snapshots for their institutions and potentially comparing and combining information for a CTSA-Network snapshot. Such an effort would facilitate monitoring enrollment for individual regions with different population benchmarks and also could provide a broader perspective of enrollment across the CTSA-Network and country. To inform other hubs or institutions about our efforts, we have described strengths and limitations of the process of developing this snapshot. We have concluded with implications and next steps to refine the ISP Snapshot and leverage its potential at Duke and beyond.

### Strengths and limitations

This Snapshot provided an overall view of the state of inclusion of special populations in clinical research at Duke. The information provided an aggregate baseline from which to identify gaps and find solutions to gaps, such as inclusion and reporting standards, increasing outreach to underserved populations (e.g., Latino and rural populations), facilitating participation across the lifespan, and ensuring that populations not represented are included (e.g., individuals identifying in non-conforming terms). Comparisons to US Census benchmarks served as a preliminary gauge about how well overall DUHS clinical research participants represent geographically relevant communities. This comparative context has been useful given regional differences across the country and world, though it remains limited in informing adequacy of enrollment relevant in the absence of specific health condition prevalence. Presentation of enrollment statistics in a data visualization format facilitated access and readability of the information for the public. Specialty tools in data visualization and guidance have become increasingly available and allowed our team to refine the ISP Dashboard without further expert consultation.

The aggregated total overall enrollment (across protocols, departments, etc.) provided a high-level view of participation in all recorded clinical research studies at this institution. This information has been a starting point and has not informed the context of what contributes to disparities in representation. The information has also been limited to protocols enrolled in OnCore by individual encounter, excluding non-interventional studies, such as observational, focus group, or survey-based research. The excluded study type may have been used to explore health behaviors and social determinants of historically understudied groups, suggesting potential bias in the OnCore enrollment information and reports. Our team has begun to examine factors which may contribute to health risks among underrepresented groups in clinical research (i.e., Black/African Americans within rural populations, and adults over 80 years) [24]. Incorporating prevalence estimates of health conditions under study would be critical to planning study samples that can inform treatment effects for affected subpopulations [25].

### Implications

Replicating snapshots, or even dashboards of enrollment across institutions could provide comparisons (e.g., large academic vs. smaller medical centers), and, in aggregate, could serve as an indicator of enrollment inclusivity across the CTSA network and nationally. Such an effort could be a useful tool to assess and monitor progress toward achieving representation in clinical research.

Towards this end, we have outlined key considerations learned throughout our experience to inform those considering creating an ISP Snapshot summary of clinical research at other institutions.

*Determine the purpose and expected audiences viewing enrollment of special populations*. The primary purpose of the ISP Snapshot has been to assess the current status of inclusion of special populations in Duke’s clinical research to inform our Core’s activities. However, the many uses of the information – from quality improvement by the institution, accountability by a sponsor, or use in community outreach and education to help address the identified disparities and underrepresentation – clearly indicate a need for an evolving tool.

*Identify data source(s) and clinical research studies included.* The ISP Snapshot, similar to Langford’s efforts to characterize clinical research across another large academic medical institution [16], leveraged an institutional-level clinical research management system designed to help monitor enrollment and other administrative records. CRMSs have not been available online at every institution, or may not have established compliance and oversight procedures ensuring that enrollment information is entered or up-to-date. Also, CRMSs have not always included all clinical research studies at an institution. While Duke’s CRMS has included the majority of clinical research studies (e.g., all funders), excluded studies (i.e., international, non-interventional, survey, focus group, and observational studies) would be anticipated to bias any OnCore-based reports. Identifying which studies are included in the data source would be an important step in assessing usefulness or limitations of any reports.

*Identify and collaborate with key stakeholders*. Because the data source(s) likely cross multiple departments, collaborating with institutional leadership and key stakeholders would be needed to determine the landscape, and to access and understand the available clinical research enrollment data. Working with other departments to create a public-facing report on data and information which has been under their control and management could elicit some degree of resistance. Communicating intentions and actions and recognizing distinct purposes in using the enrollment information (i.e., clinical research management oversight vs. making enrollment demographics broadly accessible) would be critical to ensure effective collaborations. In our experience, changes in the CRMS system reporting system during the course of developing the snapshot delayed the evolution of the snapshot, and required changes in our plans and project course. Establishing the complementary rather than competing goals and uses of the information and communication helped ensure that the ISP Snapshot/Dashboard development came to fruition.

*Operationally define variables for special populations.* To define special populations, consider how these groups have been categorized operationally in common data elements across datasets, research, or other administrative uses. Our study revealed the need for consistency among common data elements, or at the least, very clear specification of how data are accounted for (unknown value vs. missing value) or coded or consolidated (e.g., racial and/or ethnic groups, multiple racial groups, “other race” categories). Additionally, cutoffs and coding procedures for age (e.g., children, older adults) and rurality should be standardized based on research or policy. Identifying reporting variables that do not adequately reflect special populations further provides opportunities for change. Limited values for reporting *Sex* as binary only exclude reporting of any non-conforming gender identity, despite increased awareness of health care needs for this group. Developing the snapshot has prompted changes in how Duke gathers and reports information. OnCore reporting has been updated to incorporate additional non-binary and non-conforming options. The recent Office of Management and Budget Directive update [26] requiring that race and ethnicity be combined into a single reporting variable (e.g., non-Hispanic Asian) has stimulated additional discussions about aligning with this approach.

*Determine relevant benchmarks.* The ISP Snapshot uses US Census data benchmarks to assess whether our institution was enrolling special populations in clinical research with appropriate representation from the communities we serve. Another relevant benchmark of interest to administrative leadership is comparing research enrollment demographics to that of the institution’s clinical population. Finally, determining target enrollment in protocols must incorporate disease prevalence of health conditions being studied to ensure the relevance of evidence generated for population sub-groups most affected.

## Conclusions

Developing this ISP Snapshot has highlighted the potential of reporting and examining enrollment demographics within an institution. Many institutions have used reporting tools and clinical research management systems to monitor enrollment for quality assurance and monitoring of clinical research operations. However, the potential utility could extend beyond these applications. Monitoring enrollment at an institutional level might offer an understanding of how the institution’s enrollment demographics compare with published evidence on aggregated clinical trial disparities [27], effects of past events such as the COVID-19 pandemic on enrollment [2,28,29]or potential impacts of technology advances on clinical research [30]. Developing ways to incorporate disease prevalence with ISP Dashboard enrollment demographics could enhance investigators’ enrollment planning and monitoring of studies. Leveraging enrollment information in community outreach efforts could inform and engage those underrepresented to encourage their participation in clinical research toward health equity.
